# Supplementary material for: Gate-controlled skyrmion and domain wall chirality
Source: Nat Commun. 2022 Sep 7;13:5257. doi: 10.1038/s41467-022-32959-w (PMC9452545; doi:10.1038/s41467-022-32959-w)
Supplement: Supplementary file 1 — Supplementary Information [file 41467_2022_32959_MOESM1_ESM.pdf]

# Supplementary informations : Gate-Controlled Skyrmion and Domain Wall Chirality

Charles-Elie Fillion,[1] Johanna Fischer,[1] Raj Kumar,[1] Aymen Fassatoui,[2] Stefania Pizzini,[2] Laurent Ranno,[2] Djoudi Ourdani,[3] Mohamed Belmeguenai,[3] Yves Roussigné,[3] Salim-Mourad Chérif,[3] Stéphane Auffret,[1] Isabelle Joumard,[1] Olivier Boulle,[1] Gilles Gaudin,[1] Liliana Buda-Prejbeanu,[1] Claire Baraduc,[1] and Hélène Béa[1,4]

[1]*Univ. Grenoble Alpes, CEA, CNRS, Spintec, 38000 Grenoble, France*
[2]*Univ. Grenoble Alpes, CNRS, Néel Institute, Grenoble, France*
[3]*Laboratoire des Sciences des Procédés et des Matériaux (LSPM), Villetaneuse, France*
[4]*Institut Universitaire de France (IUF)*

## I. MATERIAL-DEPENDENT INVERSION OF iDMI SIGN AND CHIRALITY

The oxidation gradient at the top FeCoB/$TaO_x$ interface leads to a variation of the magnetic parameters along the top-Ta wedge. Notably, we observed that the current-induced motion (CIM) direction of magnetic skyrmions and chiral DWs is submitted to an inversion along the top Ta wedge. In Supplementary Fig. 1a, a visible-light image shows a top-view of the multilayer with the array of ITO electrodes, highlighted with a color that depends on the CIM direction measured close to it. An electrode highlighted in red corresponds to a CIM direction along the current density, while an electrode highlighted in blue corresponds to an opposite CIM direction, as represented in Supplementary Fig. 1(b,c). Between these two regions, nearly no CIM has been observed while injecting current density, corresponding to black-highlighted electrodes in Supplementary Fig. 1a.

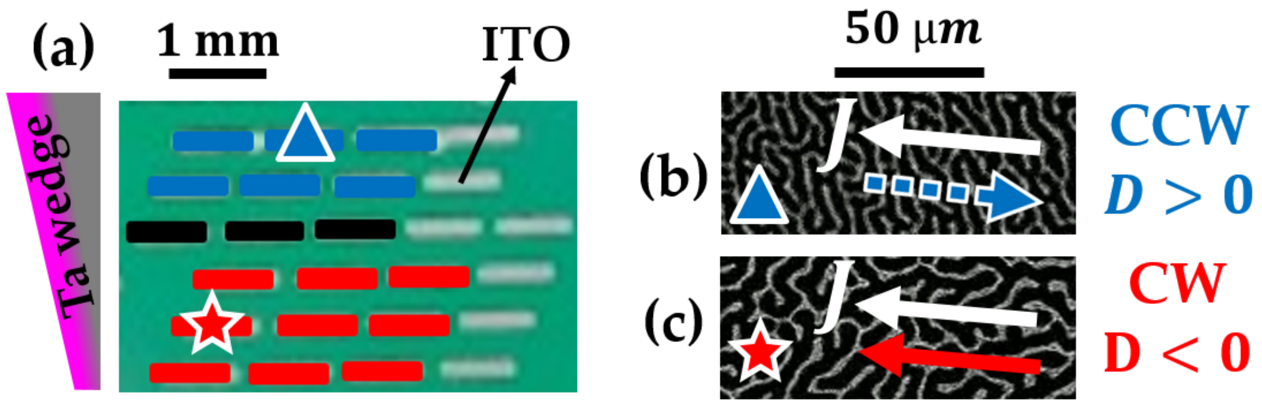


Supplementary Fig. 1: (a) Top-view of the sample in visible light. The top-Ta wedge direction is indicated on the left. The multilayer appears in green. ITO electrodes appear as grey rectangles, highlighted in a color that depends on the local chirality: red for CW chirality and blue for CCW chirality. (b)-(c) p-MOKE snapshots taken in each region, whose corresponding videos are SV8 and SV9. The current direction (depicted by the white arrow) is the same for the two snapshots, only the CIM direction changes, indicating a chirality inversion along the top-Ta-wedge.

A clear trend appears on the sample, as shown on Supplementary Fig. 1a. For thicker top-Ta layer the CIM direction is opposite to the current density (see Supplementary Fig. 1b and Supplementary Video SV8) while it is along the current density for thinner top-Ta layer (see Supplementary Fig. 1c and Supplementary Video SV9). Such observation could result either from an inversion of the iDMI sign or from an inversion of the spin accumulation sign at the bottom Ta/FeCoB interface, the latter being observed in Pt/Co/$GdO_x$[1]. As shown in Fig. 1c of main text, direct measurement of the iDMI constant through Brillouin Light Scattering (BLS) technique show that this inversion of motion direction is due to an inversion of the iDMI constant.

These observations shows that the inversion of CIM direction for DWs and skyrmions originates from an inversion of the iDMI sign. Moreover, it shows that iDMI amplitude and sign depends the oxidation state of the top FeCoB/$TaO_x$ interface. Such material-dependance for iDMI has been reported in other studies[2] with similar multilayers. Our observations show that for thinner Ta layer CW DWs are stabilized while for thicker Ta

layer they are CCW. It allowed us to study the voltage effect either starting from a CW or a CCW chirality and to show that in both cases, a gate voltage with appropriate sign can reverse the chirality (see supplementary section V).

We estimated the top-Ta thickness in the region where the chirality switch is observed around $t_{Ta} \simeq 0.92$ nm (according to deposition profile and localization on the sample). Thus, linear interpolation gives a typical order of magnitude of our iDMI around 10 $\mu$J.m$^{-2}$. This very weak value is explained by the proximity to the location of the iDMI sign inversion.

## II. DETERMINATION OF THE CURRENT DENSITY

In order to estimate the current density everywhere in the bottom Ta layer (thickness $t$, resistivity $\rho$), we elaborated a simple model where a charge current is flowing between two point-contact electrodes (resistivity $\rho_0 \ll \rho$, see Supplementary Fig. 2a). One electrode feeds charges in the Ta layer (the source, red circle on Supplementary Fig. 2b) while the other evacuates them (the sink, black circle on Supplementary Fig. 2b). The charges coming from the source are distributed over a partial-sphere (surface $2\pi Rt$) limited by the thickness of the Ta layer (represented by the dotted line in Supplementary Fig. 2a). For the model we consider only the steady-state (charge distribution at equilibrium) so that $\vec{\nabla}.\vec{J} = 0$. By simply using the Gauss theorem one gets the electric field $E = \pm\frac{\rho I}{2\pi Rt}$ where the sign stands for respectively a source and a sink. An applied current is obtained when charges circulate from the source to the sink. Then, by putting a source at $x = -\frac{L}{2}$ and a sink at $x = +\frac{L}{2}$, one can obtain the current density $\vec{J}(x,y)$ through $\vec{J} = \frac{1}{\rho}\vec{E}$, and one gets :

$$\vec{J} = \frac{I}{2\pi t}\left[\left(\frac{(x-\frac{L}{2})}{\left(x-\frac{L}{2}\right)^2+y^2} - \frac{(x+\frac{L}{2})}{\left(x+\frac{L}{2}\right)^2+y^2}\right)\vec{u_x} + \left(\frac{y}{\left(x-\frac{L}{2}\right)^2+y^2} - \frac{y}{\left(x+\frac{L}{2}\right)^2+y^2}\right)\vec{u_y}\right]$$

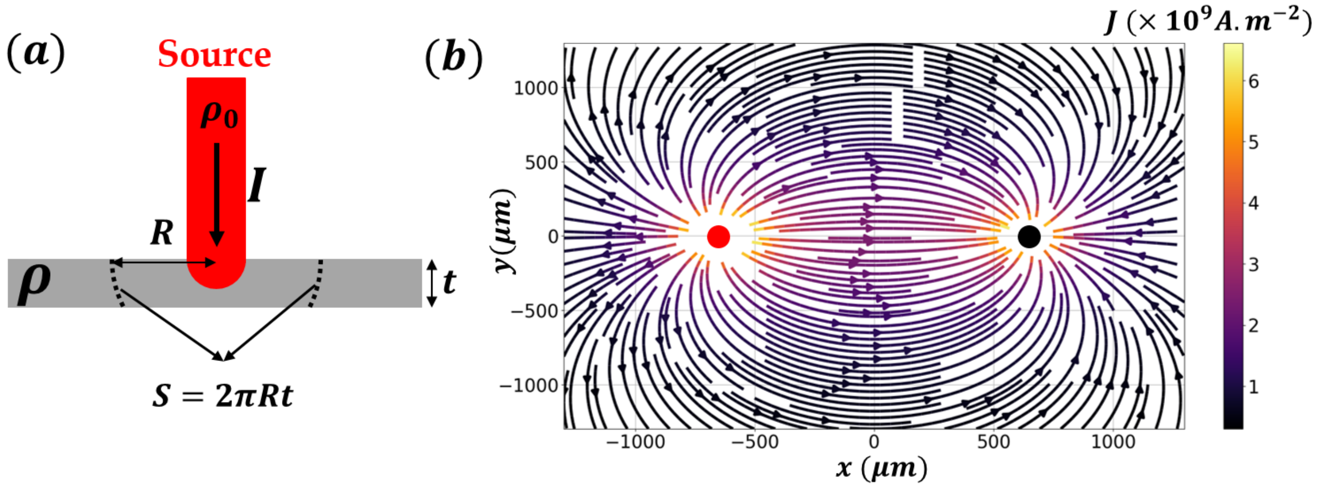


Supplementary Fig. 2: (a) Schematic representation of the cross-section geometry used for the model. The source is feeding charges that are distributed over the partial sphere defined by the dashed lines. Then, by adding a sink to the source, the current density and direction is obtained (for $I = 15\ mA$, $L = 1300\ \mu m$ and $t = 3\ nm$, typical experimental parameters), as shown in (b) where the source is depicted by the red circle and the sink by the black circle.

Then, using the experimental distance between the source and sink ($L$), injected current ($I$) and bottom Ta thickness ($t$), the current density is determined using this simple model and the average location of the skyrmion bubbles or labyrinthine DWs compared to the current density distribution. For example, in Fig. 1 of main text, the experimental parameters are $L = 1.3\ mm$, $I = 15\ mA$ and $t_{Ta} = 3\ nm$, and give the resulting current density shown in Supplementary Fig. 2b. Using this, we can extract the typical current density in the experiment region $J \simeq 5 \times 10^9\ A.m^{-2}$. Applying this method to estimate the current density in Fig. 2 of main text leads to the same order of magnitude.

## III. EFFECT OF THE THICKNESS WEDGES ON THE DOMAIN WALL AND SKYRMION MOTION DIRECTION

The wedge geometry leads to the variation of material parameters that could be at the origin of a preferential DWs motion direction. In this section, we tested the effect of the two wedges by injecting the current density along each of them with the two current polarities (see Supplementary Fig. 3a). In any case the DWs move in the direction of the current density, evidencing that the wedges have no influence on the DW or skyrmion motion direction (see Supplementary Fig. 3b and Supplementary video SV12). Thus, the DW and skyrmion motion direction is the result of the current direction associated to the DW chirality, as explained in the main text. Thus, if the current direction remains the same while a CIM direction is observed, it is the signature of an inversion of the DW chirality.
To conclude, we have shown on one hand that the weak gradient of material parameters does not influence the DW motion direction. Secondly, our gate voltage application is not expected to induce drastic change in these gradient since it is homogeneous over the ITO electrode.

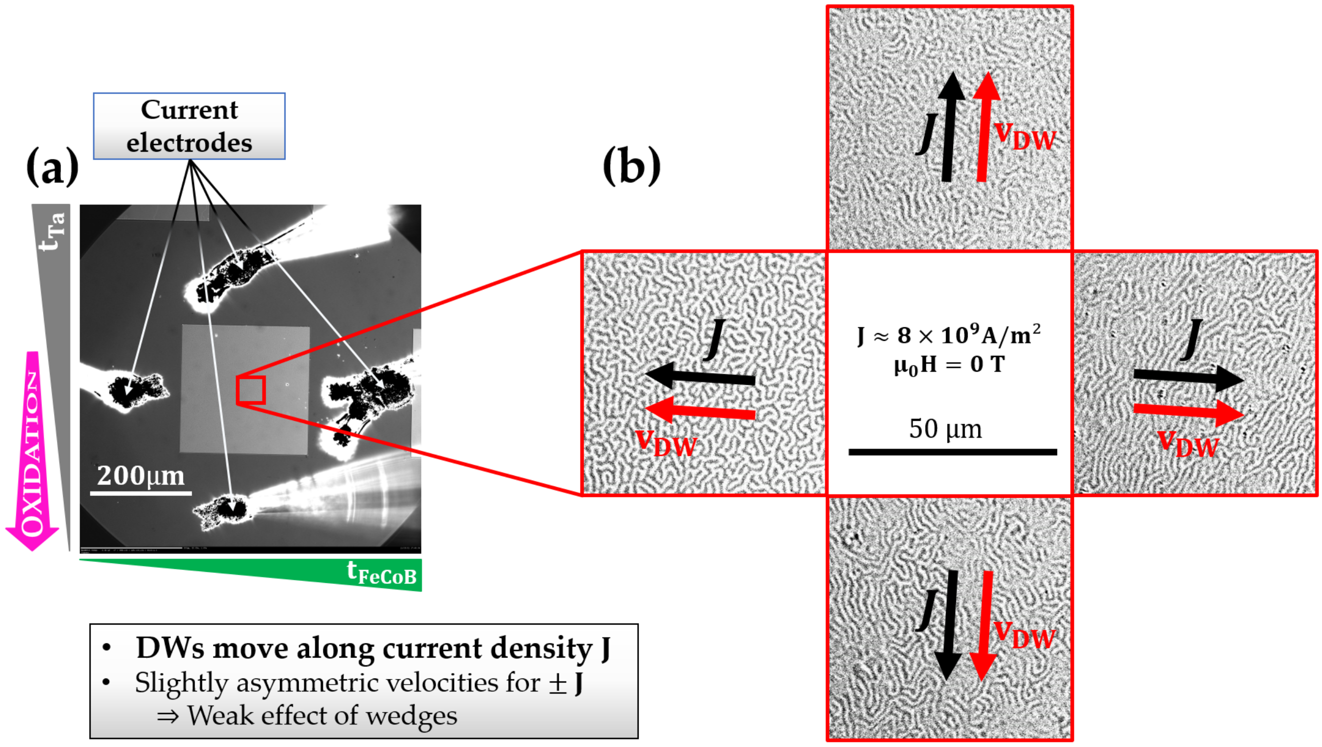


Supplementary Fig. 3: **Study of the effect of the wedge on the current induced motion direction :** (a) cross-shaped geometry of the electrical connections for current density injection. It allows four current direction, along both wedges with the two polarities. In (b) the current density is depicted by a black arrow and the DW motion direction by a red arrow.

## IV. SKYRMIONS TRAJECTORY INVERSION UNDER THE APPLICATION OF A GATE VOLTAGE

In this section we propose some additionnal details about the current induced motion of skyrmions under the application of $V_g = 0$ and $V_g > 0$, as shown in Fig. 1 of main text. From Supplementary videos SV1 and SV2, taken under the MOKE microscope, we followed the position of each moving skyrmion to extract their trajectories. It allows a better visualization of the inversion of motion direction under the application of a positive gate voltage. Moreover, the strong pinning to which the skyrmions are submitted is made clearly visible since their motion is not straight line but hopping-type trajectories with an average direction along the current density or in the opposite direction, depending on their chirality.

On Supplementary Fig. 4, the trajectories of the moving skyrmions from Fig. 1 of main text are shown. The trajectory of each skyrmion is represented as a colored line between a starting point (surrounded by a cricle) and a

ending point (surrounding by a star). On Supplementary Fig. 4(a-b) the trajectories are superimposed to the first frame of the MOKE microscope movie, on which we can see a skyrmion in each circle. For a better visualization, the trajectories are represented alone on a graph below the MOKE image (see Supplementary Fig. 2(c,d)). The ITO electrode contour is depicted by a gray line, and the direction of the current density by a black arrow. Thus, it is clear that in the initial state, skyrmions are moving in the direction of the current density, with an average velocity of 13.5 $\mu$m.s$^{-1}$ (see Supplementary Fig. 4(a,c)). Moreover, skyrmions cross the ITO edges without difficulty, revealing the continuity in the magnetic state below and around the ITO. Under the application of a positive gate voltage, the current induced motion direction is inverted, with an average velocity of $-3.2$ $\mu$m.s$^{-1}$ (see Supplementary Fig. 4(b,d)). However, some skyrmions remain totally pinned and some of them are hopping around in the bottom right corner. This might be explained by the anisotropy difference induced by the gate voltage between under and around the ITO. Skyrmions are submitted to a force, acting in the vicinity of the ITO edges and directed from the high anisotropy region (around ITO) to the low anisotropy region (under ITO)[3].

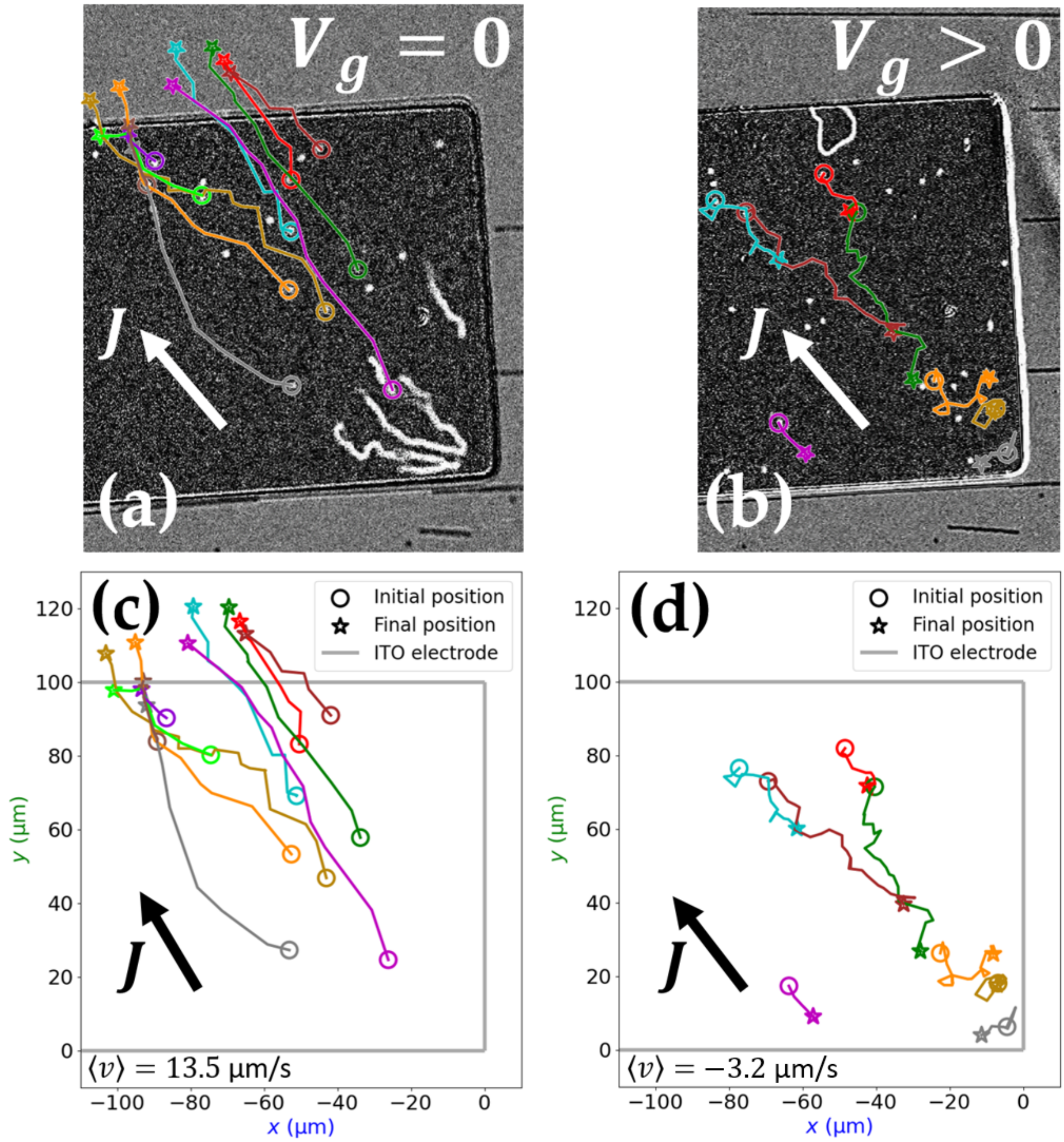


Supplementary Fig. 4: (a,c) (resp. (b,d)) Trajectory of magnetic skyrmions from Fig. 1 of main text under zero gate voltage (resp positive gate voltage). In (a) (resp. (b)), the trajectories are superimposed to MOKE microscope image of the initial position of each skyrmion (surrounded by circles). For clarity, the trajectories are represented alone in (c) (resp. (d)), where the gray rectangle corresponds to the ITO contour and the black arrow to the current density.

In Supplementary Fig. 5 we propose to show results of the same experiment on another electrode in close proximity to the one of Supplementary Fig. 4. The larger number of skyrmions in this experiment allows for more statistics and confidence in the effect. Similarly to the experiment presented above, the initial chirality is CW both below and around the ITO, since skyrmions are moving along the current density in these two regions (see Supplementary Fig. 5(a,c), and Supplementary video SV11). However, there is a small difference in the magnetic state below the ITO electrode and around it due to a previous small voltage application on the ITO electrode. Once again, the application of a positive gate voltage leads to the inversion of the current induced motion under the ITO, signature of the inversion of the skyrmions chirality (see Supplementary Fig. 5(b,d), and Suplementary video SV12). The skyrmions close to the ITO border are repealed and move along the edge. It leads to a convergent motion of the skyrmions in

the bottom left corner of the ITO, leading to an accumulation of skyrmion as it can be seen on Supplementary Fig. 5b).

It is interesting to notice that some skyrmions have common parts of trajectories. It can be explained by the presence of a local pinning or low anisotropy site in which the skyrmions passing nearby are attracted.

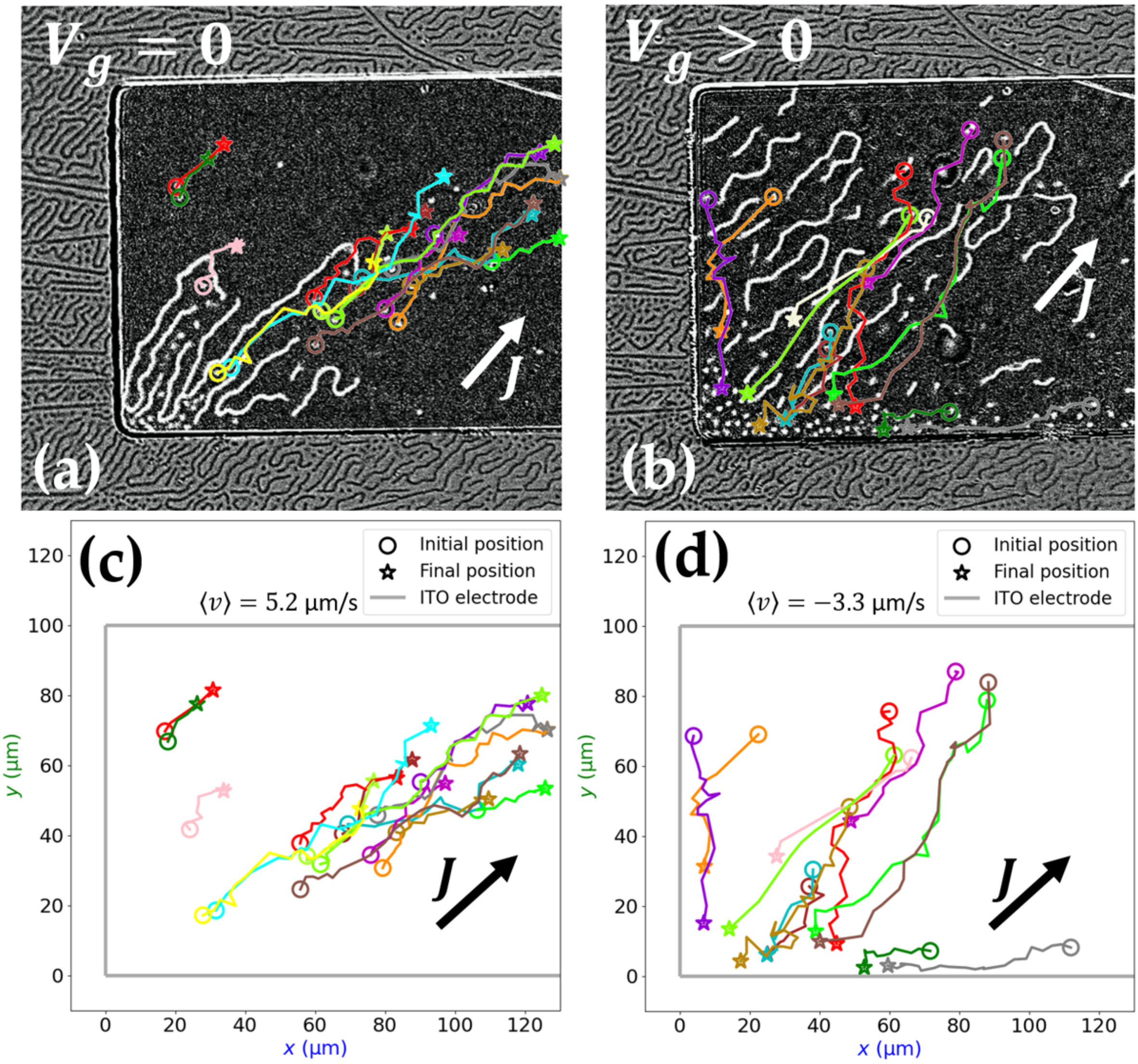


Supplementary Fig. 5: (a,c) (resp. (b,d)) Trajectory of magnetic skyrmions under another electrode with a larger number of skyrmions under zero gate voltage (resp positive gate voltage). In (a) (resp. (b)), the trajectories are superimposed to MOKE microscope image of the initial position of each skyrmion (surrounded by circles). For clarity, the trajectories are represented alone in (c) (resp. (d)), where the gray rectangle corresponds to the ITO contour and the black arrow to the current density.

## V. PERSISTENT AND REVERSIBLE CONTROL OF CHIRALITY WITH GATE VOLTAGE, DEPENDING ON INITIAL CHIRALITY

In this part we aim at demonstrating that the voltage control of chirality is possible either starting from a CW chirality ($D < 0$, star location in Fig. 1b of main text) or from a CCW chirality ($D > 0$, triangle location in Fig. 1b of main text).
In the main text of this article, the experiments are done in the star location in Fig. 1b, where the chirality is CW before gate voltage application. In this region, we showed for both skyrmions (see Fig. 1 of main text) and labyrinthine

domains (see Fig. 2 of main text) that the application of a positive gate voltage can lead to the transition from CW to CCW chirality under the ITO electrode. This is evidenced by the inversion of the CIM direction of the DWs under the ITO.
Here, we show the converse effect starting from labyrinthine domains with a CCW chirality before gate voltage application, corresponding to the triangle location in Fig 1b of main text. The external magnetic field is switched off ($\mu_0 H_{ext} = 0$) in order to get the fully demagnetized magnetic configuration shown in Supplementary Fig. 6.

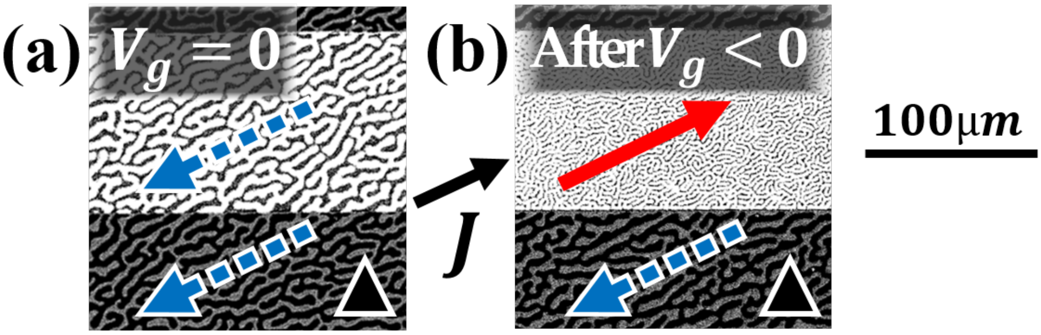


Supplementary Fig. 6: **Voltage Control of DW Chirality:** p-MOKE images of the labyrinthine configuration, under zero magnetic field. (a,b) Starting from a CCW chirality (triangle location in Fig 1b of main text), the application of a negative gate voltage on ITO can lead to a transition towards CW chirality.

The initial CCW chirality is evidenced by the CIM direction along the electron flow before applying any gate voltage on ITO electrode (dashed blue arrow on Supplementary Fig. 6a, see also Supplementary Video SV13). This time, the CIM direction under the ITO electrode is reversed (red arrow) after the application of a negative gate voltage, as shown in Supplementary Fig. 6b (see also Supplementary Video SV14, in which an external magnetic field $\mu_0 H_{ext} \simeq 30\ \mu T$ is applied in order to better distinguish the domains and their motion), which are more densily packed due to the effect of Voltage Control of Magnetic Anisotropy (VCMA). It is now along the current density, indicating a CW chirality under the ITO electrode.
In conclusion, from each side of the iDMI crossover line (the dashed line on Fig. 1b of main text), it is possible to reverse the chirality by applying a gate voltage of appropriate polarity, as schematically represented in Fig 2g of main text. Chirality is switched from CW to CCW (resp. from CCW to CW) with a positive (resp. negative) gate voltage, attributed to reduction (resp. oxidation) of the FeCoB/$TaO_x$ interface.

## VI. ANALYTICAL MODEL

In this section we present how we determined the parameters used in the analytical model in order to simulate the gate voltage application that induces a chirality inversion in FeCoB. All the parameters are summarized in Supplementary Fig. 8b.

**Determination of iDMI constant :** In supplementary section 1 we show that the iDMI depends on the location along the top-Ta wedge.
The interpolation of BLS measurement in the region where the chirality inversion has been observed ($t_{Ta} \simeq 0.92$ nm) leads to a typical order of magnitude for the iDMI constant $D \simeq 10\ \mu\text{J/m}^2$. Thus, in our calculation, the iDMI sign is inverted from $-10$ to $10\ \mu\text{J/m}^2$, through a transient $D = 0$ step that is interesting to take into account for the study of the skyrmion stability (see Supplementary Fig. 8).

**Anistropy measurements :** The anisotropy and its variation under a gate voltage were measured through hard axis hysteresis loop with the p-MOKE microscope. The data are fitted to a simple energetic model in which we consider an applied magnetic field $H_{ext}$ inclined by an angle $\alpha$ with respect to the magnetization's in-plane hard axis (see Supplementary Fig. 7b). Thus, the magnetic energy can be written as :

$$E = K_{eff} \sin^2\theta - \mu_0 H_{ext} M_S \sin(\theta + \alpha) \tag{1}$$

The first term corresponds to the uniaxial anisotropy energy and the second to the Zeeman energy. The parameter $K_{eff} = \frac{K_s}{t_{FM}} - K_d$ is the effective anisotropy constant, where $t_{FM}$ is the ferromagnetic film thickness, $K_s$ is the

interface anisotropy constant and $K_d = \frac{1}{2}\mu_0 M_S^2$ is the dipolar anisotropy constant. $\mu_0 H_{ext}$ is the applied field, $M_S$ the saturation magnetization, $\theta$ the angle between the magnetization and the easy axis and $\alpha$ the angle between the magnetic field and the hard axis (see Supplementary Fig. 7d). Then, we can obtain the equilibrium position by differentiating with respect to $\theta$. Finally, as the MOKE microscope in the polar geometry is only sensitive to $m_z$ (component along the $z$ axis of the normalized magnetization vector $\frac{\vec{M}}{M_S}$), we can write the sine and cosine in terms of $m_z$ and we finally obtain

$$\mu_0 H_{ext} = \mu_0 H_K \frac{m_z \sqrt{1-m_z^2}}{m_z \cos\alpha - \sin\alpha\sqrt{1-m_z^2}} \quad (2)$$

where $\mu_0 H_K = \frac{2K_{eff}}{M_S}$ is the anisotropy field. Thus, a fit of the experimental hard axis hysteresis curves allows to extract $\mu_0 H_K$.
Skyrmions are observed for ferromagnetic thickness close to a spin reorientation from Perpendicular Magnetic Anisotropy (PMA) to In Plane Anisotropy (IPA). In this region, the zero remanent magnetization, induced by the weak effective anisotropy, makes it impossible to use this model directly for skyrmions. However, we can take advantage of the wedge of ferromagnet to extrapolate from a region where this model is valid. For thinner ferromagnetic thickness the effective anisotropy increases since we move away from the PMA-IPA transition, and the model becomes valid when remanent magnetization is not zero. Thus we did a series of measurements, for both zero and positive gate voltage, as a function of the FeCoB thickness in order to extrapolate to the skyrmion thickness, as it is shown in Supplementary Fig. 7a. Typical raw data and their fits are shown for thinner and thicker FeCoB (resp. Supplementary Fig. 7b and Supplementary Fig. 7c.)

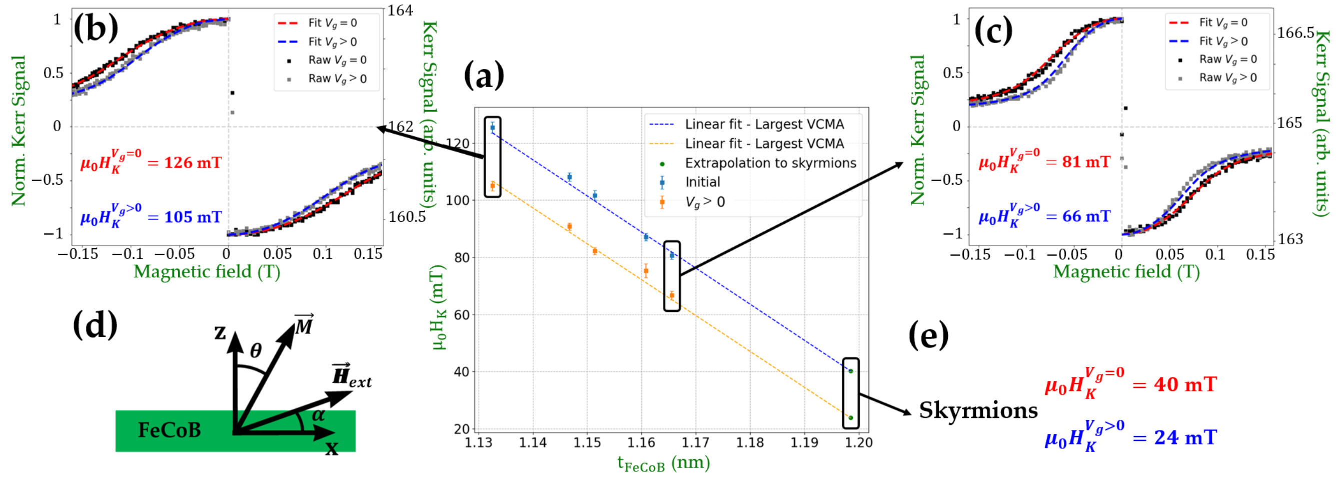


Supplementary Fig. 7: **Voltage control of magnetic anisotropy : extrapolation to skyrmions.** (a) Anisotropy field measurement versus ferromagnetic thickness before (blue) and after (orange) gate voltage application. Green points at $t_{FeCoB} \simeq 1.2$ nm corresponds to the extrapolation to skyrmion region, based on a linear fit with extreme slopes. Error bars result from the accuracy of the fit. (b,c) Raw data of hard-axis hysteresis loops and their fits with equation 1, whose geometry is shown in (d). (e) Extrapolation of VCMA to skyrmion region based on the linear fits of (a).

A linear behaviour is observed, which allowed us to extrapolate the anisotropy fields and VCMA to the thickness at which we observe skyrmions (as shown in Supplementary Fig. 7e). The presence of errobars on $\mu_0 H_K$ induces a variability on the fit slopes, and we have chosen to take the largest VCMA for skyrmions in the calculations, *ie.* the least favorable case, to ensure their stability even for large anisotropy variation. Thus, for skyrmions in our trilayer, the anisotropy field varies from 40 to 24 mT, when applying a positive gate voltage, corresponding to a decrease about $\mu_0 \Delta H_K = 16$ mT. The magnetization variation can be neglected since the variation of the Kerr signal amplitude ($\propto$ magnetization) lies within the errorbars. This is evidenced on the non-normalized scale in Supplementary Fig. 7b and 7c.

Finally, we injected iDMI and anisotropy variation in the analytical model. The results are shown in Fig. 3 of main text and reminded in Supplementary Fig. 8a. We have chosen to vary linearly the anisotropy with respect to the

iDMI value, i.e. starting from the initial $\mu_0 H_K = 40$ mT at $D < 0$, we decrease the anisotropy by $\frac{\mu_0 \Delta H_K}{2}$ at $D = 0$ and by $\mu_0 \Delta H_K$ at $D > 0$, as it is explained in Supplementary Fig. 8b. The star, triangle and diamond correspond respectively to the initial, transient and final state as defined in Fig. 3 of main text, and in Supplementary Fig. 8a.

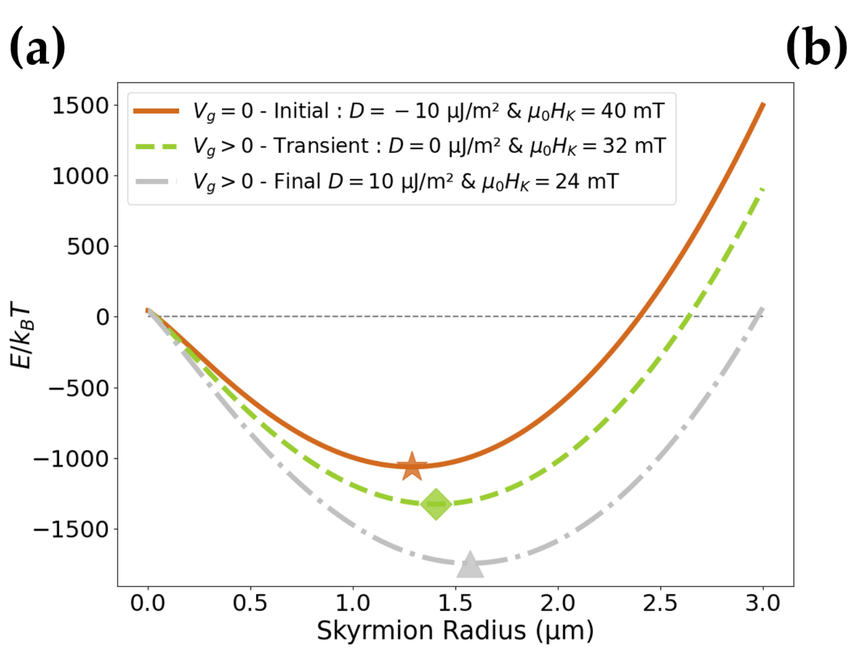


(b)

| | | |
|---|---|---|
| $M_S$ (A/m) | | $1.54 \times 10^6$ |
| $t_{DL}$ (nm) | | 0.63 |
| $t = t_{nom} - t_{DL}$ (nm) | | 0.57 |
| $A_{ex}$ (pJ/m) | | 12 |
| $\mu_0 H_a$ (µT) | | 750 |
| $\mu_0 \Delta H_K$ (mT) | | 16 |
| $\Delta K_S$ (µJ/m²) | | 10 |
| D (µJ/m²) | ★ | −10 |
| | ◆ | 0 |
| | ▲ | 10 |
| $\mu_0 H_K$ (mT) | ★ | 40 |
| | ◆ | $40 - \frac{\mu_0 \Delta H_K}{2}$ |
| | ▲ | $40 - \mu_0 \Delta H_K$ |
| $K_S$ (mJ/m²) | ★ | 0.83 |
| | ◆ | $0.83 - \frac{\Delta K_S}{2}$ |
| | ▲ | $0.83 - \Delta K_S$ |

Supplementary Fig. 8: **Chirality inversion with a gate voltage - Analytical model:** (a) Reminder of Fig. 3 of main text: Analytical calculation of energy difference (in units of $k_B T_{300K}$) between skyrmion and uniform state for FeCoB as a function of skyrmion diameter. Solid orange, dashed green and dash-dotted gray line correspond respectively to negative, zero and positive iDMI, associated to a progressive anisotropy variation as experimentally measured. (b) Summary of the parameters used in the analytical model.

The saturation magnetization and the magnetically dead layer thickness were extracted via magnetometry (both Superconducting QUantum Interference Device and Vibrating Sample Magnetometers) versus FeCoB nominal thickness and estimated respectively to $M_S = 1.54 \pm 0.06$ MA.m$^{-1}$ and $t_{DL} = 0.63 \pm 0.03$ nm. It leads to a weak 1% variation of $K_S$ from 0.83 to 0.82 mJ/m$^2$, since $t_{FeCoB} = t^{nom}_{FeCoB} - t_{DL} = 0.57$ nm, where $t^{nom}_{FeCoB}$ is the nominal thickness. The applied magnetic field has been set to $\mu_0 H_{ext} = 750$ $\mu$T opposite to the skyrmion core. Finally, the exchange stiffness has been taken to $A_{ex} = 12$ pJ/m[4].

## VII. MICROMAGNETIC SIMULATIONS

Here we give more details on the simulations of part 3 of the main text. Using Mumax3[5] we simulated an isolated skyrmion in an infinite magnetic thin film by computing the demagnetizing field from an infinite sample acting on the simulation region, a 512×512 nm region. This approximation remains valid as long as the skyrmion DW is not too close to the borders of the simulation region. The demagnetizing field is computed under Mumax3 by substracting the demagnetizing field of the 512×512 nm region with saturated magnetization (along $-\vec{z}$, where $\vec{z}$ is defined on Supplementary Fig. 9) to the one of an infinite thin film ($\vec{H}^{dem}_{\infty} = M_S \vec{z}$). The resulting demagnetizing field is set as an external field during the simulation, in addition to an homogeneous magnetic field $\mu_0 H_z = -6\ mT$. A cross-section view of the demagnetizing field is shown in Supplementary Fig. 9a (the green line in the inset depicts the location of the cross-section on the simulation region). It remains almost constant ($\mu_0 H^{dem}_z = 4.6$ mT) for any position not too close to the edges of the simulation region (it drastically increases at $\simeq 20$ nm from the edges). Moreover, the maximum skyrmion diameter is represented (double headed blue arrow), thus confirming that the demagnetizing field is roughly constant throughout the skyrmion surface. Hence, the approximation of isolated skyrmion in an infinite thin film can be considered valid. Moreover the use of a square geometry provides an additional hint of the presence of edge effects: they can be considered negligible as long as the skyrmion shape remains circular.

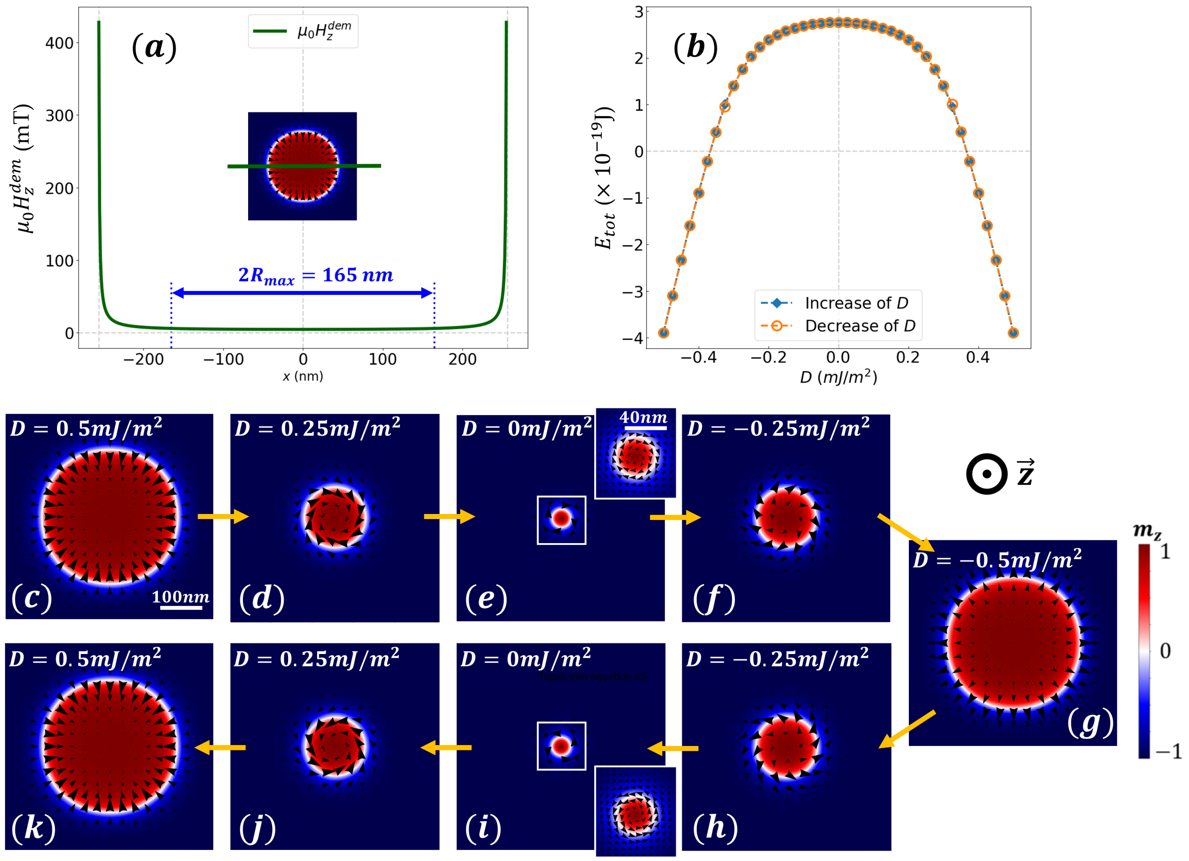


Supplementary Fig. 9: **Micromagnetic simulation:** (a) Section of the demagnetizing field at the position of the skyrmion center (as depicted by the green line in the inset). It remains almost constant over the size of the skyrmion, even for the maximum skyrmion size $R_{max}$ = 165 nm, represented by the blue arrow. (b) Total energy as a function of $D$. Energy states characterized by $D$ and -$D$ are degenerated. (c-k) Stable skyrmion for decreasing $D$ from $0.5 mJ/m^2$ to $-0.5\ mJ/m^2$ (c-g) and increasing back to $0.5\ mJ/m^2$ (g-k). A CW Bloch skyrmion is obtained for the decrease (e) while a CCW Bloch skyrmion is obtained for the increase (i). These two stable Bloch skyrmions at $D = 0$ are energetically degenerated as shown in (b).

The simulation consists in making a loop on the $D$ value and stabilizing the resulting magnetic configuration at each step. First, the $D$ value is set to $D = 0.5\ mJ/m^2$. Then, it is decreased towards $D = -0.5\ mJ/m^2$ by steps of 5%. Finally, $D$ is increased back towards $D = 0.5\ mJ/m^2$ by steps of 5%. Supplementary Figs. 9(c,e,f) correspond respectively to Figs. 4(c,b,a) of main text.
By doing so, we observed that the CCW Néel skyrmion at $D = 0.5\ mJ/m^2$ shown in Supplementary Fig. 9c is converted into a CW Néel skyrmion at $D = -0.5\ mJ/m^2$ (see Supplementary Fig. 9g), and converted back to a CCW Néel skyrmion at $D = 0.5\ mJ/m^2$ (see Supplementary Fig. 9k).
Moreover, we observed that the DW angle $\xi$ (called the helicity, as defined in Fig. 3d of main text) evolves gradually for intermediate value of $D$, between $|\xi| = 0$ (CW Néel) and $|\xi| = \pi$ (CCW Néel). Notably, we observed that $\xi$ is positive when decreasing $D$ (see Supplementary Fig. 9(d-f)) while it is negative when increasing $D$ (see Supplementary Fig. 9(h-j)). These two situations are energetically equivalent as shown in Supplementary Fig. 9b. As a result, the magnetic moments in the DW of the Bloch skyrmions obtained at $D = 0$ have opposite directions in Supplementary Fig. 9e and Supplementary Fig. 9i, and these two Bloch skyrmions are energetically degenerated (see Supplementary Fig. 9b).
Finally, an animation of the total simulation can be found in the Supplementary video SV15.

## VIII. SUPPLEMENTARY FILES

**"SV1"** : Video corresponding to Fig. 1(c,d) of main text and Supplementary Fig. 4(a,c). CIM under $V_g = 0$. The injected current is $I = 15mA$, the corresponding current density is $J \simeq 5 \times 10^9 A.m^{-2}$. ClockWise chirality for skyrmion bubbles under ITO ($D < 0$)

**"SV2"** : Video corresponding to Fig. 1(f,g) of main text and Supplementary Fig. 4(b,d). CIM under $V_g > 0$. The injected current is $I = 15mA$, the corresponding current density is $J \simeq 5 \times 10^9 A.m^{-2}$. CounterClockWise chirality for skyrmion bubbles under ITO ($D > 0$)

**"SV3"** : Video corresponding to Fig. 2a of main text. CIM under $V_g = 0$ for labyrinthine chiral DWs ($\mu_0 H_{ext} \simeq 30$ $\mu T$ in order to distinguish the domain wall motion). $I = 15mA$. ClockWise chirality for DWs under ITO ($D < 0$)

**"SV4"** : Video corresponding to Fig. 2b of main text. CIM after 90s $V_g = 3V$ voltage pulse for labyrinthine chiral DWs ($\mu_0 H_{ext} \simeq 30$ $\mu T$ in order to distinguish the domain wall motion). $I = 15mA$. CounterClockWise chirality for DWs under ITO ($D > 0$)

**"SV5"** : Video corresponding to Fig. 2c of main text. CIM after 90s $V_g = -2V$ voltage pulse for labyrinthine chiral DWs ($\mu_0 H_{ext} \simeq 30$ $\mu T$ in order to distinguish the domain wall motion). $I = 15mA$. ClockWise chirality for DWs under ITO ($D < 0$)

**"SV6"** : Video corresponding to Fig. 2d of main text. CIM after 90s $V_g = 3V$ voltage pulse for labyrinthine chiral DWs ($\mu_0 H_{ext} \simeq 30$ $\mu T$ in order to distinguish the domain wall motion). $I = 15mA$. CounterClockWise chirality for DWs under ITO ($D > 0$)

**"SV7"** : Video corresponding to Fig. 2e of main text. CIM for labyrinthine chiral DWs 2 hours after the voltage was shut down ($\mu_0 H_{ext} \simeq 30$ $\mu T$ in order to distinguish the domain wall motion). $I = 15mA$. ClockWise chirality for DWs under ITO ($D < 0$)

**"SV8"** : Video corresponding to Supplementary Fig. 1b. CIM for labyrinthine chiral DWs ($\mu_0 H_{ext} = 0$). $I = 10mA$. CounterClockWise chirality for DWs ($D > 0$)

**"SV9"** : Video corresponding to Supplementary Fig. 1c. CIM for labyrinthine chiral DWs ($\mu_0 H_{ext} = 0$). $I = 10mA$. ClockWise chirality for DWs ($D < 0$)

**"SV10"** : Video corresponding to Supplementary Fig. 3. CIM at $V_g = 0$. The injected current is $I = 18mA$, the corresponding current density is $J \simeq 8 \times 10^9 A.m^{-2}$. ClockWise chirality for DWs. ($D < 0$)

**"SV11"** : Video corresponding to Supplementary Fig. 5(a,c). CIM under $V_g = 0$. The injected current is $I = 15mA$, the corresponding current density is $J \simeq 5 \times 10^9 A.m^{-2}$. ClockWise chirality for skyrmion bubbles under ITO ($D < 0$)

**"SV12"** : Video corresponding to Supplementary Fig. 5(b,d). CIM under $V_g > 0$. The injected current is $I = 15mA$, the corresponding current density is $J \simeq 5 \times 10^9 A.m^{-2}$. CounterClockWise chirality for skyrmion bubbles under ITO ($D > 0$)

**"SV13"** : Video corresponding to Supplementary Fig. 6a. CIM under $V_g = 0$ for labyrinthine chiral DWs ($\mu_0 H_{ext} = 0$). $I = 10mA$. CounterClockWise chirality for DWs under ITO ($D > 0$)

**"SV14"** : Video corresponding to Supplementary Fig. 6b. CIM after $V_g < 0$ for labyrinthine chiral DWs ($\mu_0 H_{ext} \simeq 30$ $\mu T$ in order to better distinguish the domain wall motion). $I = 10mA$. ClockWise chirality for DWs under ITO ($D < 0$)

**"SV15"** : Animation of the chirality reversal as simulated under MuMax3, corresponding to Supplementary Fig. 9.

[1] R. Mishra, F. Mahfouzi, D. Kumar, K. Cai, M. Chen, X. Qiu, N. Kioussis, and H. Yang, "Electric-field control of spin accumulation direction for spin-orbit torques," Nature Communications, vol. 10, p. 248, Dec. 2019.
[2] M. Arora, J. M. Shaw, and H. T. Nembach, "Variation of sign and magnitude of the Dzyaloshinskii-Moriya interaction of a ferromagnet with an oxide interface," Physical Review B, vol. 101, p. 054421, Feb. 2020.
[3] E. Tamura, C. Liu, S. Miki, J. Cho, M. Goto, H. Nomura, R. Nakatani, and Y. Suzuki, "Skyrmion confinement and dynamics in tracks patterned with magnetic anisotropy: theory and simulations," arXiv:2005.04860, May 2020.
[4] T. Srivastava, M. Schott, R. Juge, V. Kryzakova, M. Belmeguenai, Y. Roussigné, A. Bernand-Mantel, L. Ranno, S. Pizzini, S.-M. Chérif, A. Stashkevich, S. Auffret, O. Boulle, G. Gaudin, M. Chshiev, C. Baraduc, and H. Béa, "Large-Voltage Tuning of Dzyaloshinskii-Moriya Interactions: A Route toward Dynamic Control of Skyrmion Chirality," Nano Letters, vol. 18, pp. 4871–4877, Aug. 2018.
[5] A. Vansteenkiste, J. Leliaert, M. Dvornik, M. Helsen, F. Garcia-Sanchez, and B. Van Waeyenberge, "The design and verification of MuMax3," AIP Advances, vol. 4, p. 107133, Oct. 2014.
