# Supplementary material for: Gate-controlled skyrmion and domain wall chirality
Source: Nat Commun. 2022 Sep 7;13:5257. doi: 10.1038/s41467-022-32959-w (PMC9452545; doi:10.1038/s41467-022-32959-w)
Supplement: Supplementary file 2 — Description of Additional Supplementary Files [file 41467_2022_32959_MOESM2_ESM.pdf]

Title: Supplementary Movie 1
Description: corresponding to Fig. 1(c,d) of main text and Supplementary Fig. 4(a,c). CIM under V_g = 0. The injected current is I = 15mA, the corresponding current density is $J \approx 5 \times 10^9$ A/m^2. ClockWise chirality for skyrmion bubbles under ITO (D < 0)

Title: Supplementary Movie 2
Description: corresponding to Fig. 1(f,g) of main text and Supplementary Fig. 4(b,d). CIM under V_g > 0. The injected current is I = 15mA, the corresponding current density is is $J \approx 5 \times 10^9$ A/m^2. CounterClockWise chirality for skyrmion bubbles under ITO (D > 0)

Title: Supplementary Movie 3
Description: corresponding to Fig. 2a of main text. CIM under V_g = 0 for labyrinthine chiral DWs ($\mu_0H_{ext} \approx 30$ µT in order to distinguish the domain wall motion). I = 15mA. ClockWise chirality for DWs under ITO (D < 0)

Title: Supplementary Movie 4
Description: corresponding to Fig. 2b of main text. CIM after 90s V_g = 3V voltage pulse for labyrinthine chiral DWs ($\mu_0H_{ext} \approx 30$ µT in order to distinguish the domain wall motion). I = 15mA. CounterClockWise chirality for DWs under ITO (D > 0)

Title: Supplementary Movie 5
Description: corresponding to Fig. 2c of main text. CIM after 90s V_g = -2V voltage pulse for labyrinthine chiral DWs ($\mu_0H_{ext} \approx 30$ µT in order to distinguish the domain wall motion). I = 15mA. ClockWise chirality for DWs under ITO (D < 0)

Title: Supplementary Movie 6
Description: corresponding to Fig. 2d of main text. CIM after 90s V_g = 3V voltage pulse for labyrinthine chiral DWs ($\mu_0H_{ext} \approx 30$ µT in order to distinguish the domain wall motion). I = 15mA. CounterClockWise chirality for DWs under ITO (D > 0)

Title: Supplementary Movie 7
Description: corresponding to Fig. 2e of main text. CIM for labyrinthine chiral DWs 2 hours after the voltage was shut down ($\mu_0H_{ext} \approx 30$ µT in order to distinguish the domains). I = 15mA. ClockWise chirality for DWs under ITO (D < 0)

Title: Supplementary Movie 8
Description: corresponding to Supplementary Fig. 1b. CIM for labyrinthine chiral DWs (μ_0H_{ext} = 0). I = 10mA. CounterClockWise chirality for DWs (D > 0)

Title: Supplementary Movie 9
Description: corresponding to Supplementary Fig. 1c. CIM for labyrinthine chiral DWs (μ_0H_{ext} = 0). I = 10mA. ClockWise chirality for DWs (D < 0)

Title: Supplementary Movie 10
Description: corresponding to Supplementary Fig. 3. CIM at V_g = 0. The injected current is I = 18mA, the corresponding current density is $J \approx 8 \times 10^9$ A/m^2. ClockWise chirality for DWs. (D < 0)

Title: Supplementary Movie 11
Description: corresponding to Supplementary Fig. 5(a,c). CIM under V_g = 0. The injected current is I = 15mA, the corresponding current density is $J \approx 5 \times 10^9$ A/m^2. ClockWise chirality for skyrmion bubbles under ITO (D < 0)

Title: Supplementary Movie 12
Description: corresponding to Supplementary Fig. 5(b,d). CIM under $V_g > 0$. The injected current is I = 15mA, the corresponding current density is $J \approx 5 \times 10^9$ A/m^2. CounterClockWise chirality for skyrmion bubbles under ITO (D > 0)

Title: Supplementary Movie 13
Description: corresponding to Supplementary Fig. 6a. CIM under $V_g = 0$ for labyrinthine chiral DWs ($\mu_0 H_{ext} = 0$). I = 10mA. CounterClockWise chirality for DWs under ITO (D > 0)

Title: Supplementary Movie 14
Description: corresponding to Supplementary Fig. 6b. CIM after $V_g < 0$ for labyrinthine chiral DWs ($\mu_0 H_{ext} \approx 30$ µT in order to better distinguish the domain wall motion). I = 10mA. ClockWise chirality for DWs under ITO (D < 0)

Title: Supplementary Movie 15
Description: Animation of the chirality reversal as simulated under MuMax3, corresponding to Supplementary Fig. 9.
